# Matching method between nanoparticle displacement agent size and pore throat in low permeability reservoir

**DOI:** 10.3389/fchem.2023.1289271

**Published:** 2023-10-26

**Authors:** Tianjiang Wu, Yanhong Zhao, Yichi Zhang, Zhixiao Li, Junwei Su

**Affiliations:** ^1^ School of Human Settlements and Civil Engineering of Xi’an Jiaotong University, Xi’an, China; ^2^ Oil and Gas Technology Research Institute of Changqing Oilfield, China National Petroleum Corporation, Xi’an, China; ^3^ Research Institute of Xi’an Changqing Chemical Group Co., Ltd., of Changqing Oilfield, China National Petroleum Corporation, Xi’an, China; ^4^ Oil Production Plant NO 11 of Changqing Oilfield, China National Petroleum Corporation, Qingyang, China; ^5^ Oil Production Plant NO 10 of Changqing Oilfield, China National Petroleum Corporation, Qingyang, China

**Keywords:** nanoparticle, size matching, low permeability reservoir, advantage channel, enhanced oil recovery

## Abstract

Nano-particles possess desirable attributes such as small particle size, excellent injectivity, and migration performance, making them highly compatible and adaptable for addressing the water flooding requirements of the low-permeability oil reservoir. When selecting an oil displacement agent for enhancing water flooding and improving oil recovery, factors such as injectivity and migration need to be carefully considered. In this study, through a comprehensive analysis of the mechanism and technical characteristics of nano-particle oil displacement agents, the plugging and profile control mechanisms recognized by the mainstream of nano-particles are elucidated. By examining various elements including outcrop fractures, natural micro-fractures, artificial support fractures, and dynamic monitoring data, a reevaluation of the dominant channel scale governing water drive in low permeability reservoirs is conducted, thereby defining the target entities for profile control and flooding operations. Drawing upon Darcy’s percolation law and leveraging enhanced oil recovery techniques based on the classical Kozeny equation, a profile control and flooding mechanism is proposed that focuses on increasing the specific surface area of polymer particles while simultaneously reducing reservoir permeability. This innovative approach establishes a novel matching method between nano-polymer particles and the diverse media found within the reservoir. Lastly, the application of nanoparticle flooding technology in Changqing Oilfield is presented, highlighting its practical implementation and benefits.

## 1 Introduction

Petroleum is a strategically significant resource that plays a vital role in the national economy and ensures energy security at the national level (Alvarado and Manrique, 2010; Chu and Majumdar, 2012). As of 2022, China’s reliance on foreign crude oil had reached 71.3%. A significant portion of oil and gas reserves in China are stored in low-permeability reservoirs. The newly discovered and developed oilfields primarily consist of low-permeability, ultra-low permeability, and shale reservoirs. These types of reservoirs are characterized by their low permeability, small pore throats, limited reserves, pronounced heterogeneity, and considerable extraction challenges (Xu et al., 2023). During the water injection development process, the physical properties and heterogeneity of these reservoirs have a profound impact (Al-Shalabi and Ghosh, 2016; Bakhshian et al., 2020). This influence often leads to the formation of preferential water flow pathways, which expedite the rise in water cut and decline in oil production from wells (Lenormand et al., 1988; Zhang et al., 2011; Hu et al., 2020). Consequently, non-uniform displacement of injected water emerges as a critical factor limiting both well productivity and the ultimate recovery rate of the reservoir (Lei et al., 2022; Liu and Wang, 2022). Profile control and flooding technology presents an effective solution for impeding the preferential water flow channels. By implementing this technology, fluid flow diversion can be achieved, leading to an expanded coverage of water drive within the reservoir. This technique has found widespread application in enhancing water drive efficiency, controlling water encroachment, and maintaining stable oil production in water injection development reservoirs (Sze et al., 2003; Dai et al., 2012; Zhao et al., 2015).

The utilization of nanoparticle profile control and flooding technology has emerged as a prominent area of research for achieving comprehensive profile control in order to enhance water flooding efficiency and improve oil recovery in low-permeability reservoirs. This technology has gained considerable attention due to its distinctive features, including small particle size, exceptional injection and migration capabilities, and substantial effectiveness in displacing oil within the reservoir (Fu et al., 2011; Shen et al., 2022; Zhang et al., 2022; Lei et al., 2023). The Institute of French Petroleum Research (IFP) has prepared a water-soluble polyacrylamide microsphere that can significantly reduce water content in oil wells. Nalco Company synthesized a polymer nanosphere that can hydrolyze and expand, and conducted on-site experiments in Koluel Kaike and other oil fields, achieving good oil increasing effects. Wang et al. prepared micrometer polymer microspheres that can reduce the permeability of the effluent layer (Wang et al., 2020). Zhang et al. prepared nano and micro polymer microspheres, which achieved industrial processing and production (Zhang et al., 2022). They conducted on-site experiments in oil fields such as Shengli, North China, Daqing, and Changqing, and achieved good oil increase and precipitation effects. Lei et al. synthesized submicron and micrometer sized polymer microspheres and conducted experiments on high permeability reservoirs in Shengli Island, improving oil recovery (Lei et al., 2022; 2023). Zhu et al. conducted on-site injection tests on polymer microspheres in Daqing Oilfield (Zhu et al., 2022; 2023).

Currently, there remains a considerable level of dispute regarding the underlying mechanism by which nano-particle oil displacement agents enhance oil recovery. The primary area of focus revolves around whether the dominant mechanism is primarily driven by capillary forces or plugging mechanisms. Yao et al. believed that the main mechanism of polymer microspheres for profile control and flooding is bridging and sealing, mainly manifested as selective sealing of large pores, causing liquid flow to turn and oil droplets to converge into oil flow after sealing (Yao et al., 2012; 2013; 2014). Zhu et al. analyzed and found that polymer microsphere particles exhibit two types of non-uniform flow in microscale flow, and established a mathematical model for nano micron polymer particle dispersion system flooding considering the distribution characteristics of non-uniform concentration (Zhu et al., 2022). Wu et al. envisioned the Brownian motion of nano polymer microspheres to form flow resistance on the water phase of high permeability bands, reduce the fingering and channeling of injected water, and ultimately drive out the relatively low permeability layer of crude oil (Wu et al., 2023). The above is only subjective speculation and imagination, lacking sufficient theoretical explanations and corresponding experimental verification. At present, the main research method for the mechanism of microsphere oil displacement is to observe the detachment effect of nanoparticles on oil droplets through microscopic porous media chip visualization technology. Wasan et al. proposed the possibility of using the structural separation pressure of nanoparticles in the field of oil displacement, and conducted oil droplet detachment experiments using surfactant micelles on glass plates (Wasan and Nikolov, 2003). Xie et al. used the Lattice Boltzmann method to study the oil displacement process of polymer microsphere dispersion system (Xie et al., 2018; 2020). Analysis suggests that increasing the viscosity or elastic modulus of dispersed phase can further improve oil recovery rate. Lu et al. described the changes in capillary force during the process of nano microsphere infiltration displacement through capillary bundle experiments, and studied the velocity dependence of capillary pressure and the displacement dynamics of continuous oil and gas water flow (Lu et al., 2023).

According to the existing research reports, the mechanism of nano-particle plugging and profile control is a mainstream view that is generally accepted. From the perspective of particle application type, polymer nanoparticles play a leading role. When the ratio of polymer microsphere particle size to pore throat diameter is between 1.20 and 1.50, polymer microsphere has good migration ability and sealing effect. Yao et al. believe that when the ratio of microsphere particle size to pore throat diameter is 1.42, the microsphere sealing rate and maximum deformation migration pressure gradient reach their maximum (Yao et al., 2013). Dai et al. studied the matching law and deep regulation mechanism between elastic gel dispersion and pore throat, and proposed that when the matching coefficient is optimized between 0.20 and 0.31, the gel dispersion particles have good injection performance and regulation ability in the rock core (Dai et al., 2012). A mathematical model of microsphere particle size and reservoir throat was established using Weibull distribution function, and corrected it by integrating reservoir heterogeneity and microsphere flow characteristics. James et al. believe that the optimal ratio of particle size to pore throat is 1/10 (James et al., 2003).

The nano polymer particle is generally formed by copolymerization of acrylamide, acrylic acid and cross-linked monomer reverse phase micro lotion, with small initial particle size and has good suspension dispersion and viscoelasticity in the water phase, it is continuously hydrated and expanded after being injected into the formation. During migration, a single particle with relatively large particle size expands and blocks the pore throat, while several small particles accumulate and block through expansion. Under the action of pressure, a large number of particles constantly undergo hydration expansion, plugging, deformation, and re-plugging in the pore throat to achieve a large adjustment of the water flooding flow field and expansion of the swept volume.

Changqing Oilfield is widely recognized as a representative reservoir with low porosity and permeability, characterized by narrow pore throats and limited permeability. When it comes to selecting suitable techniques for improving water flooding and enhancing oil recovery in such reservoirs, factors such as injection and migration play crucial roles. The distinct technical characteristics of nano-polymer particles align well with the requirements for enhancing water flooding in Changqing reservoir. Consequently, since 2010, Changqing Oilfield has dedicated efforts towards conducting technical research and testing focused on improving oil recovery through profile control and flooding utilizing nano-polymer particles. In recent years, considering the unique geomorphic conditions and reservoir demands within the loess plateau, Changqing Oilfield has successfully developed a range of nano-polymer particles with specific particle sizes of 100 nm, 300 nm, and 800 nm. Leveraging an online centralized injection process implemented at the water injection station, this technology has been extensively applied in an industrial context across over 20,000 wells. As a result, it has become the primary technical approach for enhancing water flooding and optimizing oil recovery within Changqing Oilfield.

Utilizing the specific characteristics of low-permeability reservoirs in Changqing Oilfield as a foundation, this study conducts an analysis of the mechanism and technical attributes of nano-particle oil displacement agents. Moreover, it elucidates the recognized plugging and profile control mechanisms prevailing in the realm of nano-particles. By investigating outcrop fractures, natural micro-fractures, artificially supported fractures, and dynamic monitoring data, a fresh comprehension is attained regarding the scale of advantageous water drive channels in low-permeability reservoirs. Furthermore, this research clarifies the target entities for water drive control. Building upon the principles of Darcy’s percolation law and the classical Kozeny equation, a comprehensive technical analysis is performed to enhance oil recovery. Within this framework, a mechanism is proposed that involves augmenting the specific surface area of polymer particles while concurrently reducing reservoir permeability, and established a mathematical relationship between different particle sizes and plugging rate of nano polymer microspheres. Therefore, a novel approach is established for aligning nano-polymer particles with porous media within the reservoir. Finally, this paper introduces the practical application of nano-particle oil displacement technology within Changqing Oilfield, showcasing its implementation and impact in the field.

## 2 Identification of dominant channel in low permeability reservoir

### 2.1 Traditional classification of dominant seepage channels

The fundamental perspective of the nano-particle plugging mechanism revolves around the concept that the initial diameter of polymer active particle pellets, prior to water absorption and expansion, is at the nanometer scale. This size is significantly smaller than the diameter of pore throats within the formation, which typically range in the micron scale. During the initial stages of injecting polymer active particle pellets for profile control and flooding, these pellets smoothly absorb water within the injection system and gradually expand as the injected water reaches the formation depth. Subsequently, a process of temporary plugging, breakthrough, temporary plugging, and breakthrough of water flow channels takes place. This process primarily targets entry into high permeability layers and large pore throats to induce a plugging effect. Simultaneously, water within the dispersion system enters low permeability layers and small pore throats, directly interacting with the remaining oil contained within them. Thus, the combined action of polymer particle gel micelles and water enables the coordinated and synchronized execution of nanoparticle profile control and flooding. In summary, nano-polymer particles exhibit the characteristics of “injecting, blocking, and moving.” However, to effectively employ nanoparticle technology for profile control and flooding, it becomes essential to address the challenge of matching nanoparticles with the target reservoir. In this matching process, understanding the size of the dominant seepage channel in the low permeability reservoir becomes critical for selecting the appropriate nanoparticle injection size.

**FIGURE 1:**
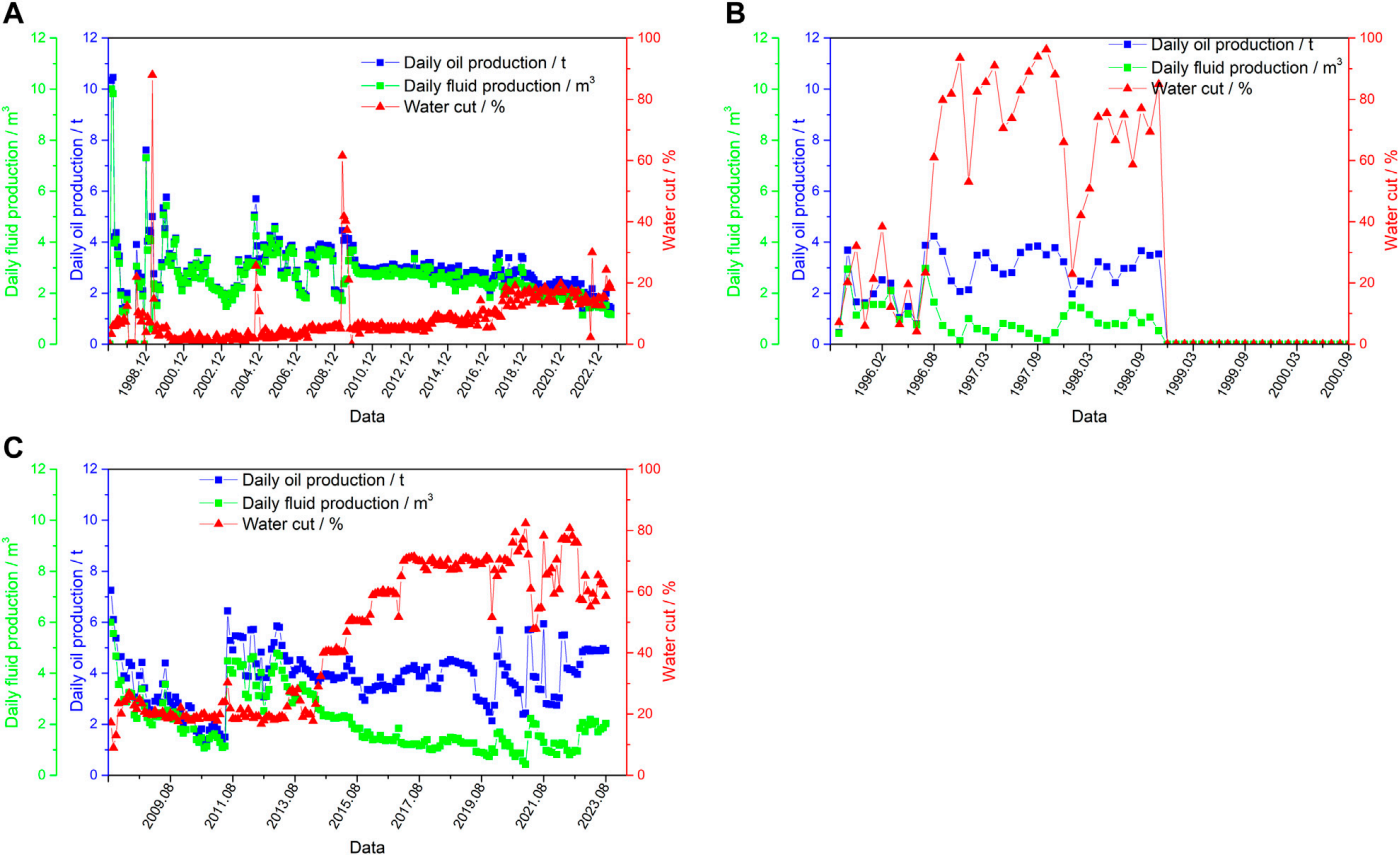
Three typical dominant seepage channel types **(A)** pore water breakthrough, **(B)** fracture water breakthrough, **(C)** pore-fracture water breakthrough.

Three types of water breakthrough in oil wells are summarized in production practice: pore water breakthrough (Figure 1A), fracture water breakthrough (Figure 1B) and pore-fracture water breakthrough (Figure 1C). Corresponding to three typical types of traditional dominant seepage channels.

**FIGURE 2:**
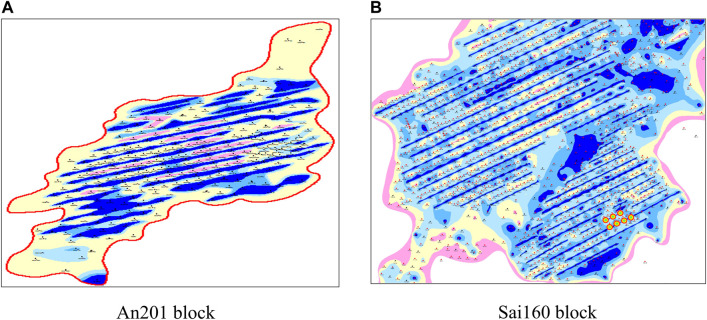
Characteristics of the through waterline shown in the water content contour map, **(A)** An201 block and **(B)** Sai160 block.

The water line reflected by the water content contour map is generally considered as a through crack. There are 12 through-waterlines in An 201 block (Figure 2A) and 24 through-waterlines in Sai 160 block (Figure 2B). The water breakthrough ratio of main directional well is high (20.9%), and the effect of lateral well is slow. Pressure maintenance level <60%. In fact, contour map is a common form to reflect the characteristic trend, which only represents the water content trend, but does not represent the existence of large-scale dominant channels or cracks in the underground. Therefore, high water cut or flooding at the wellhead is not equivalent to the existence of large-scale dominant channels or through fractures in the formation.

### 2.2 New insight of dominant seepage channels

**FIGURE 3:**
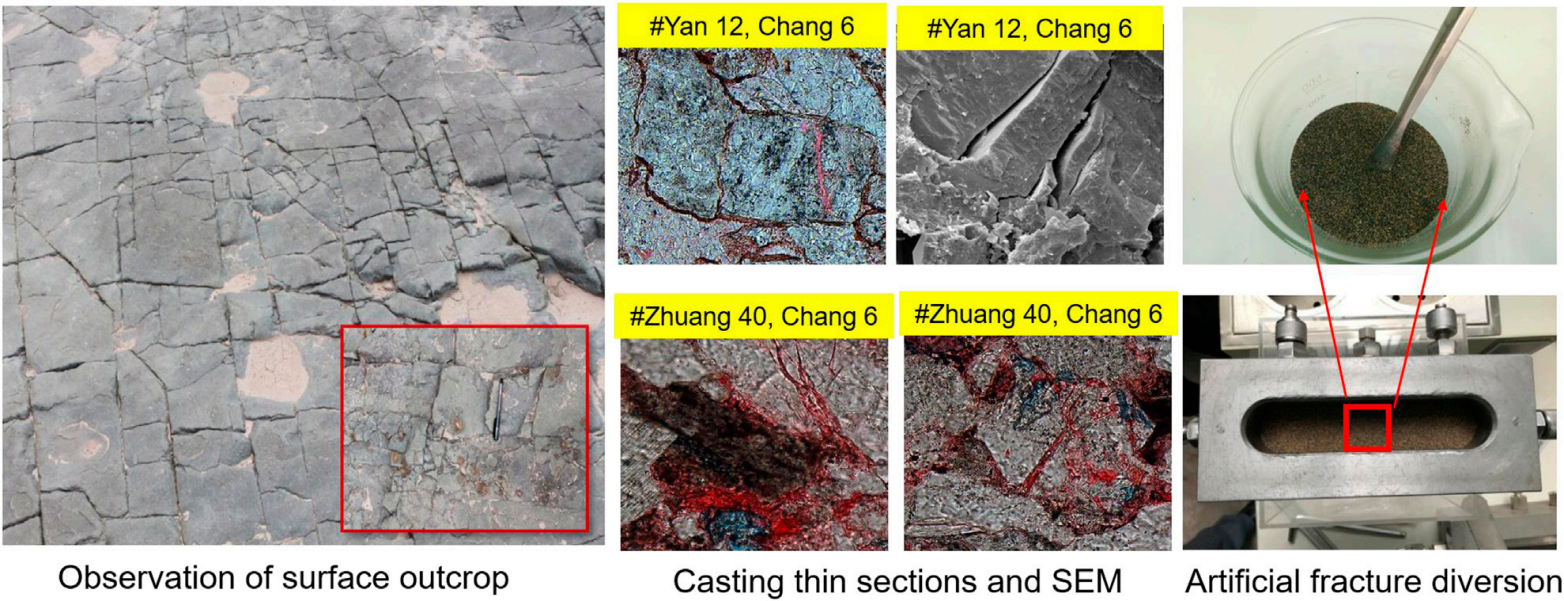
Comparison of three visible crack scales.

The Triassic dominant channel is mainly composed of dynamic fractures, micro-fractures and artificial fractures, and micro-fractures are the main targets for plugging. There are three observable fracture scales (as shown in Figure 3): outcrop fracture, microfracture and artificial diversion fracture. The first type: Two groups of east-west and south-north natural fractures are developed in the upper sandstone of Chang 6 in Dongjiahe area, with fracture spacing of 15–30 cm and fracture opening is millimeter. The second method: casting thin section and scanning electron microscope observation showed that the micro-crack opening of Chang 6 was 5–8 μm. Which were mainly connected through pore throat. Third, the fracturing fracture permeability simulated by artificial fracture diversion can reach 10–40 μm^2^, its corresponding closing pressure is 10–25 MPa, and the approximate estimated pore throat diameter is 100–400 μm.

The first kind of understanding is the most common. Its technical orientation is to plug with large ball above millimeter level and high viscosity profile control and displacement agent, which is neither feasible nor economical in fact. However, under formation pressure (above 15–20 MPa), fractures mainly exist in closed form. Further consideration, what is the significance of using quartz sand or ceramist as proppant in artificial fracturing in terms of the long-term stable production cycle of oil wells if the underground fracture is in the form of an open fracture?

The dominant channel of Jurassic is mainly composed of high permeability layers. There are almost no dynamic fractures in the Jurassic. The permeability of Jurassic high permeability layer is about 300 × 10^−3^ μm^2^, and the corresponding pore throat diameter is about 7–15 μm.

The oil layer from the water injection well to the oil well is divided into three zones successively: dynamic fracture, microfracture and fracturing fracture. The pore volume controlled by dynamic fracture area accounts for 2% of the control volume of well group, while the pore volume controlled by microfracture area accounts for more than 80% of the control volume of well group. Therefore, reducing the permeability of the middle of the injection-production and producing the remaining oil enrichment area determine that the nano-polymer particles need to have good injectivity and migration.

## 3 Nanoparticle size matching method

### 3.1 Technical analysis of reducing the permeability of dominant channels

Based on the principle of seepage mechanics, the characteristics of injection-production seepage flow field changed by profile control and flooding were analyzed. The general expression of Darcy’s linear flow is:

$$Q=A\cdot v=A\cdot \frac{K\Delta p}{uL}$$ (1)

where *Q* is flow, m³/s, *A* is the cross-sectional area, m^2^, *v* is the flow velocity, m/s, *K* is permeability, μm^2^, *u* is fluid viscosity, Pa·s, Δ*p* is seepage pressure difference, Pa, *L* is the seepage length, m.

It can be seen from Eq. 1 that for constant pressure production, displacement pressure difference is constant, and the control of production is essentially the control of mobility. The technical objective of profile control and flooding to improve oil recovery is to reduce the permeability of the dominant flow channel through the presence of profile control and flooding agent at the depth of the reservoir. The viscosity of the displacement medium is increased in the short term in the process of profile control and flooding, but the ultimate goal is to reduce the permeability of the dominant channel by long-term residence of the profile control and flooding agent.

In the early study of reservoir physics, foreign scholars obtained the mathematical relationship between permeability and petrological parameters, that is, permeability value can be determined indirectly through petrological parameters. For sandstone reservoir, the permeability has the following relationship:

$$K=\frac{\phi^{3}}{2\tau^{2}S^{2}}\times 10^{8}$$ (2)

where *Φ* is porosity, %, *τ* is tortuosity, constant, the value in this article is 3, *S* is specific surface, m^−1^.

From Eq. 2, permeability is directly proportional to porosity and inversely proportional to tortuosity and specific surface area. The total internal surface area of pores per unit volume of rock is the specific surface. Obviously, the smaller the particle, the larger the specific surface. For low-permeability reservoirs with relatively small water flooding seepage velocity, a large number of nanoparticles are widely distributed in the pore throat network of rocks. By relying on the contact accumulation, retention and plugging of nanoparticles, the overall plugging effect is showed up, and the specific surface is greatly increased, thus reducing the permeability, and achieving the purpose of reducing the seepage velocity and changing the direction of water flooding.

The matching method of nano-polymer particle has changed from the theory of pore throat plugging to increasing specific surface and reducing permeability, which has solved the long-standing technical contradiction between injection and plugging, and makes it possible to match the injection agent with the formation; Through the migration of nano-polymer particles to the center, through the material modification, van der Waals force and other functions to achieve adsorption and aggregation, and finally achieve the goal of profile control and flooding to expand the swept volume.

### 3.2 Theoretical calculation of plugging rate

If the nanoparticles are regarded as spheres, the volume and surface area of a single particle can be expressed by Eq. 3 and 4:

$$V=\frac{4}{3}\pi r^{3}$$ (3)

$$A=4\pi r^{2}$$ (4)

where *V* is particle volume, m³, *A* is particle surface area, m^2^, *r* is particle radius, m.

At present, there are two kinds of nanoparticles for profile control and flooding: inorganic particles and organic particles. Nanopolymer microspheres are organic particles synthesized from polyacrylamide, which have swelling properties and can undergo hydration expansion when exposed to water. During the implementation of nano polymer microsphere flooding in the mine, hydration and expansion will occur when nano polymer microsphere particles are injected into the reservoir from the wellhead. At the same time, it should be noted that nano inorganic particles do not exhibit hydration expansion characteristics.

Therefore, by introducing the expansion factor, the volume and surface area of a single nano polymer microsphere particle can be represented by Eq. 5 and 6:

$$V_{E}=\frac{4}{3}\pi {\left(r\times \frac{E_{m}}{2}\right)}^{3}$$ (5)

$$A_{E}=4\pi {\left(r\times \frac{E_{m}}{2}\right)}^{2}$$ (6)

Where $E_{m}$ is the expansion factor, generally taken as 3–7 times. The specific value will be explained in the subsequent actual calculation process. $V_{E}$ is particle volume with expansion factor, m³, $A_{E}$ is particle surface area with expansion factor, m^2^.

For nanoparticles, the seepage resistance in pore space is small, and the particles mainly migrate to the throat where seepage resistance is large to play the role of profile control and flooding. Compared with pore space, throat plays a decisive role in permeability, so that porosity has less influence on surface calculation. Before conducting relevant theoretical derivation and calculations, there are two assumptions that need to be explained, firstly, the aggregation mode of nanoparticles is point contact arrangement and stacking, and secondly, the pore throat space filled by the injection of nanoparticle solution into the formation. For nanoparticles of a certain mass, prepare a nanoparticle water solution according to a certain mass concentration, the pore volume *V*_*p*_ affected by the nanoparticles is the particle solution volume *V*_*q*_, and the corresponding rock volume, specific surface area and internal surface area can be expressed as follows:

$$V_{s}=\frac{M}{\rho }$$ (7)

$$S_{s}=\sqrt{\frac{\phi^{3}}{2\tau^{2}K}}$$ (8)

$$A_{s}=V_{s}\cdot S_{s}$$ (9)

The number and total surface area of the corresponding nanoparticles are respectively expressed as:

$$N=\frac{M}{\rho V}$$ (10)

$$A_{T}=A\cdot N$$ (11)

The specific surface of the pore volume affected by the injection of nanoparticles is:

$$S_{T}=\frac{A_{T}+A_{s}}{V_{s}}$$ (12)

where *V*_*s*_ is rock volume, m³; *M* is the particle mass, Kg; *ρ* is particle density, Kg/m³; *S*_*s*_ is the specific surface of rock, m^−1^; *A*_*s*_ is the internal surface area, m^2^; *N* is the number of particles; *A*_*T*_ is the total surface area of particles, m^2^; *S*_*T*_ is the specific surface after particle injection, m^−1^.

Substitute the specific surface value of pore throat space after nanoparticle injection calculated by Eq. 12 into Eq. 2 to obtain the permeability calculation equation after nanoparticle profile control and flooding:

$$K_{q}=\frac{\phi^{3}\cdot {V_{s}}^{2}}{2{\left[\tau \cdot \left(A_{T}+A_{s}\right)\right]}^{2}}\times 10^{8}$$ (13)

where *K*_*q*_ is the permeability after particle profile control and flooding, μm^2^.

It can be seen that the general calculation equation of plugging performance of profile control and displacement agent is:

$$\eta =\frac{K-K_{q}}{K}$$ (14)

After substituting Eq. 13 into Eq. 14, it can be obtained that:

$$\eta =\frac{K-\frac{\phi^{3}\cdot {V_{s}}^{2}}{2{\left[\tau \cdot \left(A_{T}+A_{s}\right)\right]}^{2}}\times 10^{8}}{K}$$ (15)

where *η* is the plugging rate of nanoparticle, %.

The permeability values before and after nanoparticle profile control and flooding were substituted into Eq. 15 to obtain the plugging rate of particle profile control and flooding. For the characteristics of the continuous hydration and expansion of nanoparticles over time, it is necessary to consider the change of the particle size of nanoparticles, and calculate the plugging rate corresponding to different injection stages and different reservoir residence positions of nanoparticles. Based on the reservoir parameters and the physical and chemical parameters of nanoparticles, the specific surface value of the accumulation of nanoparticles in the pore throat was calculated. According to the relationship between the petrological parameters and permeability, the permeability value after the injection of nanoparticles can be obtained, and then the plugging rate of profile control and flooding by injecting nanoparticles can be calculated.

## 4 Example calculation and experimental comparison

### 4.1 Example calculation

Chang 6 reservoir in Wangyao has an average porosity of 13.7% and an average permeability of 2.29 × 10^−3^ μm^2^, which is the earliest ultra-low permeability reservoir developed on land in China. The block has been developed by water injection for more than 20 years, with water content increase rapidly, the production decreased greatly and the uneven water flooding contradiction is prominent. The average permeability of strong water flushed zone interpreted by water flooding in inspection well is 16.3 × 10^−3^ μm^2^, the permeability of the dominant large pore channel interpreted by tracer monitoring is between (49–300) × 10^–3^ μm^2^, which is the main interval for profile control and flooding. Deep profile control and flooding of nanopolymer particles to improve water flooding has been carried out in Wangyao Block since 2015.

In order to realize the step-by-step profile control and flooding for different water flushed intervals, The injection slug of 100 nm, 300 nm and 800 nm nanoparticles were designed. The plugging rate of nanoparticle profile control and flooding is calculated according to the theoretical method of increasing specific surface area by nanoparticle profile control and flooding. The relevant parameters of nano polymer microspheres are as follows: the mass of the nanoparticles is 5,000 kg, and the density is 1.05 kg/m³, expansion factor is 3 times.

**FIGURE 4:**
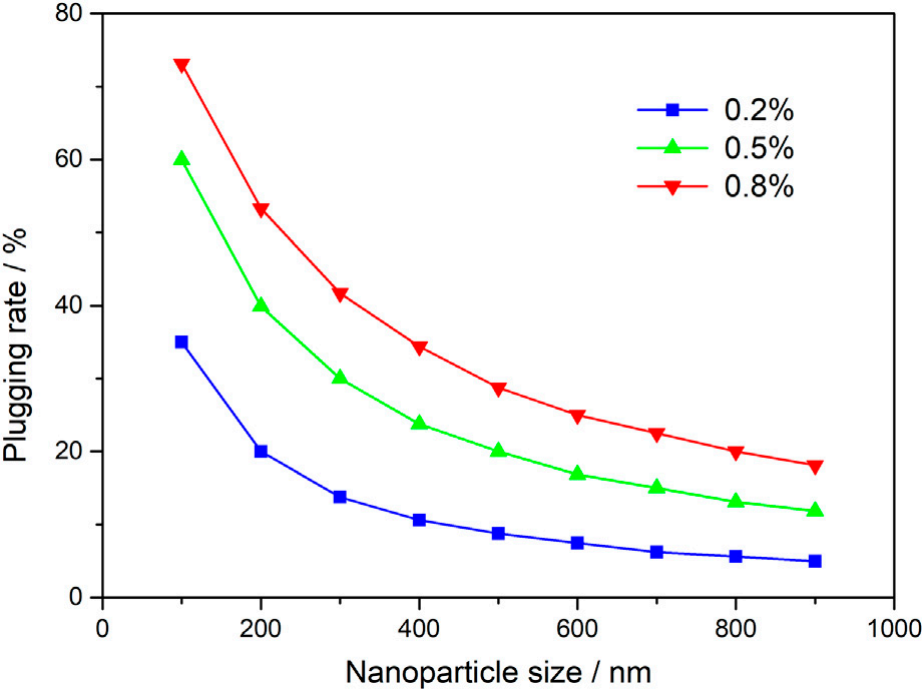
Theoretical plate of particle size and plugging rate in Wangyao densification zone.

It can be seen from Figure 4 that the smaller the particle size of nanoparticles, the greater the plugging rate calculated by the specific surface theory method, and the higher the mass concentration, the higher the plugging rate of nanoparticles. For 100 nm nanoparticles, the theoretical plugging rate is less than 40% when the mass concentration is 0.2%, 60% when the mass concentration is 0.5%, and 78% when the mass concentration is 0.8%. The analysis shows that the mechanism of nanoparticle profile control and flooding is different from that of traditional gel system. The gel is a continuous phase system, which can achieve the effect of plugging by filling pore and throat through integral gelling, while the nanoparticle is a dispersed phase system, which relies on the aggregation of nanoparticles to form plugging. The higher the mass concentration, the greater the aggregation density of nanoparticles, and the more obvious the increase of specific surface effect. With the continuous injection of nanoparticles, the nanoparticles continuously break through the pore throat and migrate, and finally stay in the deep formation to achieve the effect of profile control and flooding step by step. Under the condition of low permeability reservoirs, the migration ability and shear resistance of the gel are not as good as that of the nanoparticle system, while the increase of the specific surface area of the nanoparticle particles can produce a strong overall plugging effect.

**FIGURE 5:**
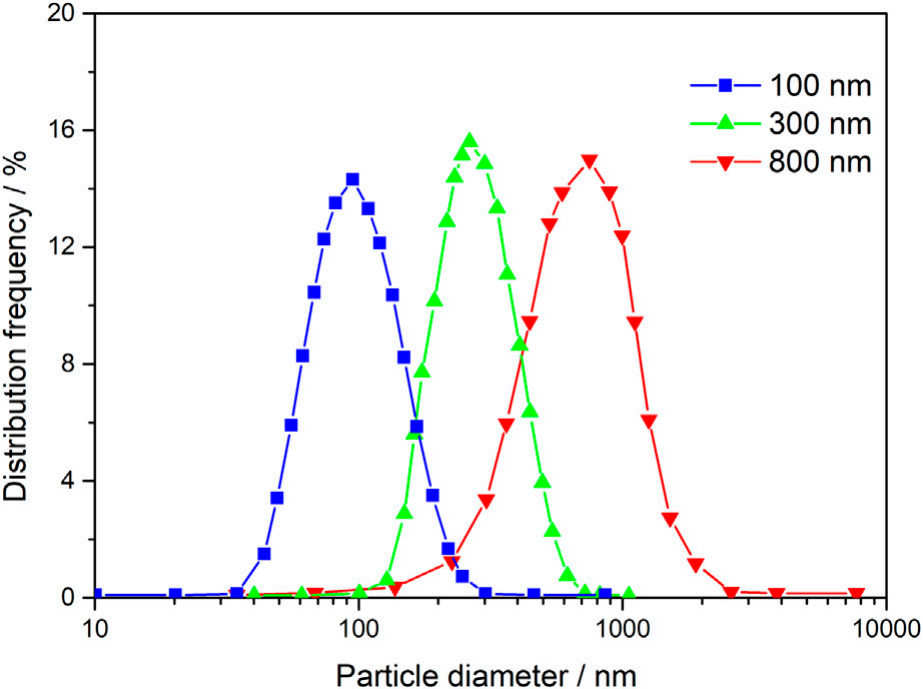
Initial particle size distribution of 100, 300, and 800 nm nanoparticles.

The nanoparticles with size of 100, 300, and 800 nm can basically meet the matching application of ultra-low permeability, extra-low permeability, and low permeability reservoirs. The initial particle size distribution of 100 nm, 300 nm, and 800 nm nanoparticles is shown in Figure 5.

### 4.2 Comparison of NMR experiments

**FIGURE 6:**
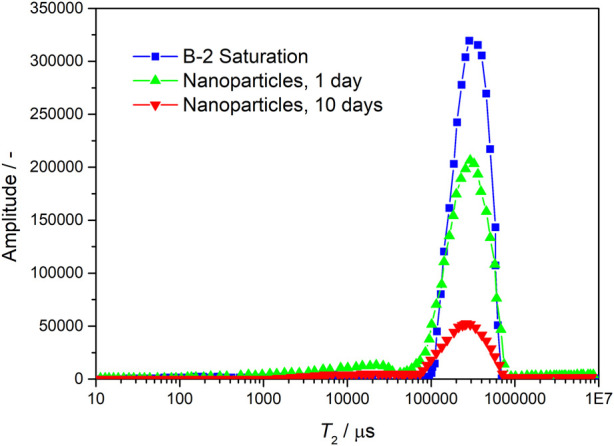
Nuclear magnetic resonance scanning results of core B-2 before and after particle displacement.

**FIGURE 7:**
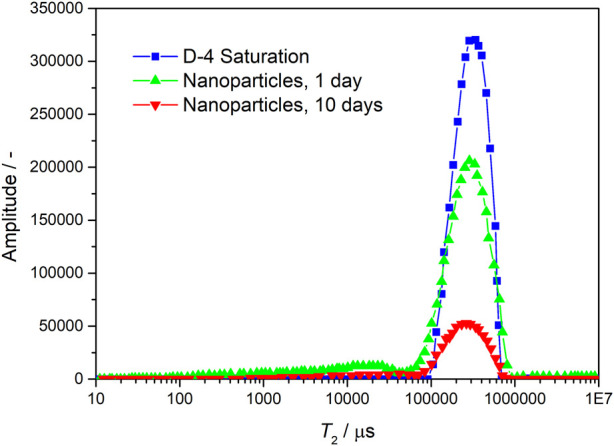
Nuclear magnetic resonance scanning results of core D-4 before and after particle displacement.

The changes of specific surface area and permeability were calculated and compared by scanning tests at different times before and after the injection of nanoparticles with NMR experiment. The larger the relaxation time and amplitude of NMR T2, the larger the pore throat scale and space, while the smaller the amplitude, the smaller the pore throat scale and space. From Figures 6, 7, it can be seen that after the injection of nanoparticles, the relaxation amplitude decreases with the prolongation of residence time, indicating that the injection of nanoparticles fills the pore throat space, resulting in a smaller pore throat time. The experiments show that the nanoparticles can increase specific surface area and reduce permeability (as shown in Table 1).

**TABLE 1:** Nuclear magnetic resonance scanning and calculation results of sand filling model before and after particle profile control and flooding.

Different stages of core	B-2 saturation	B-2 nanoparticles, 1 day	B-2 nanoparticles, 10 days	D-4 saturation	D-4 nanoparticles, 1 day	D-4 nanoparticles, 10 days
Permeability (mD)	137.9	132.7	34.79	379.2	326.1	43.65
Specific surface area (cm^2^/cm^3^)	692.3	747.8	1,378	506.6	685.2	1,493
Specific surface area amplification (%)	―	8.02	99.09	―	35.25	194.8
Permeability reduction (%)	―	3.78	74.77	―	14.02	88.49

### 4.3 Field application and effect

The understanding of the mechanism underlying nanoparticle profile control and flooding has evolved beyond pore throat matching to encompass specific surface matching. It has become evident that small particle size plays a pivotal role in achieving deep profile control and flooding with nanoparticles, thereby guiding the transition from micron-scale to nanometer-scale product designs. Through independent research and development efforts, we have successfully created a series of nanoparticle products with particle sizes of 50, 100, and 300 nm, along with corresponding technical standards. To facilitate the large-scale application of nano-polymer particles, a process model has been established, characterized by small particle size (ranging from 50 to 300 nm), low concentration (0.1%–0.2%), significant liquid volume (5,000 m^3^, adjusted based on recovery degree), and centralized injection (at water injection plants or valve blocks). This process model has effectively promoted the widespread utilization of nano-polymer particles. Between 2017 and 2023, nano-polymer particles were implemented in over 20,000 wells within Changqing Oilfield. Notably, the implementation area experienced an average reduction of 2% in the natural decline rate, a decrease of 0.3% in the water cut increase rate, and an increase of 4.2% in the dynamic recovery rate.

**FIGURE 8:**
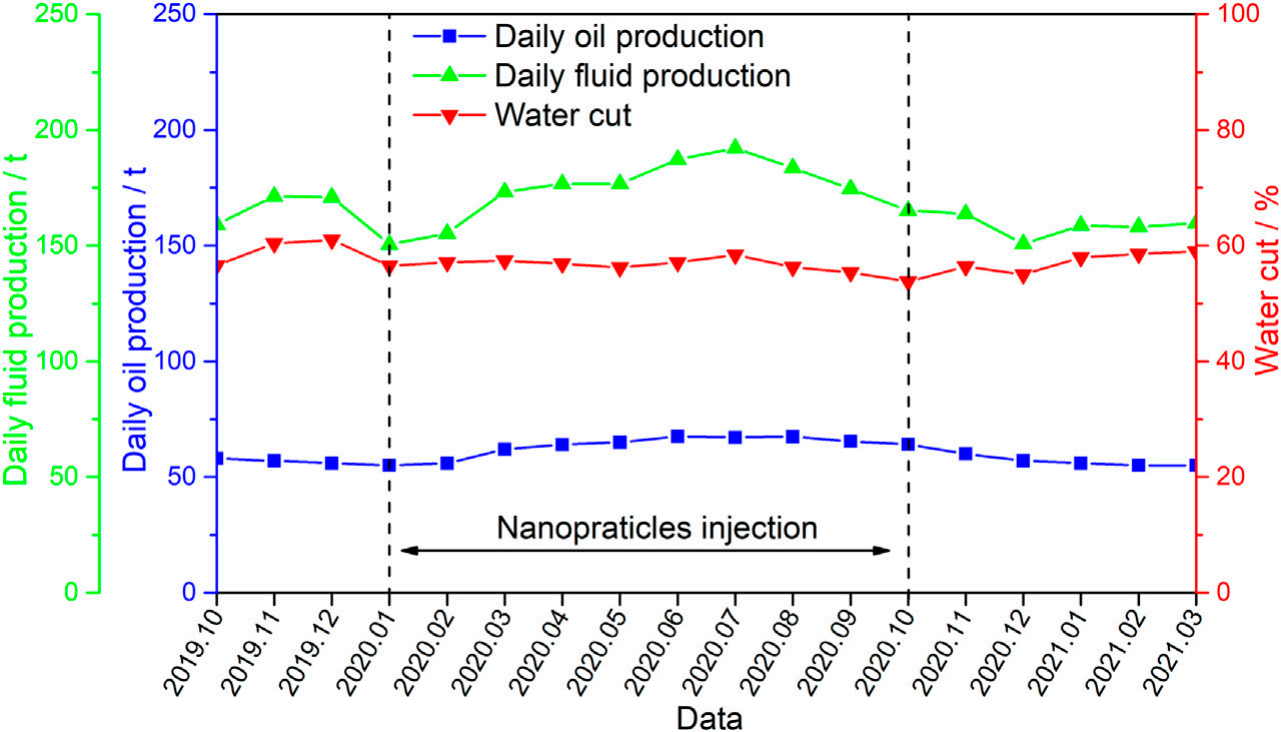
Effect of nanopolymer microspheres on flooding control in S301 block.

Typical application block: S301 is the main reservoir of Ansai Oilfield, and nano polymer microsphere particle control and flooding were implemented from January to October 2020. The particle size is 100 nm, the total injection mass is 5 t, and the injection mass concentration is 0.1%–0.2%. From Figure 8, it can be seen that after adjusting the displacement, the daily oil production increased from 55 t to 62.6 t, and the peak daily oil production reached 67.5 t. The effective period of the measures was 12 months, with a cumulative increase of 2,475 t. The water cut has also been effectively controlled.

## 5 Conclusion

The size of the dominant channel is analyzed from the change of water content in oil wells and the existence forms of fractures, which provides a foundation of technical understanding for the matching of nanoparticle size and reservoir. Based on the classical Karzny equation, the theory of expanded swept volume of nanoparticle profile control and flooding is developed, the matching method of nanoparticle size with reservoir is established, and the theoretical calculation and experimental test are compared. The new matching method guides the transition of the synthetic particle size of nano-polymer particles from microns to nanometers, and the 50–300 nm polymer particles have been developed. The enhanced oil recovery technology of nano-polymer particle profile control and flooding has achieved large-scale application in Changqing low-permeability reservoir, and has achieved good results.

## Data Availability

The raw data supporting the conclusion of this article will be made available by the authors, without undue reservation.
